# Inter-ankle Systolic Blood Pressure Difference Is a Marker of Increased Fasting Blood-Glucose in Asian Pregnant Women

**DOI:** 10.3389/fendo.2022.842254

**Published:** 2022-05-31

**Authors:** Ruo Zhang, Hema Viswambharan, Chew Weng Cheng, Malgorzata Anna Garstka, Kirti Kain

**Affiliations:** ^1^ Department of Endocrinology, The Second Affiliated Hospital of Xi’an Jiaotong University, Xi’an, China; ^2^ Leeds Institute of Cardiovascular and Metabolic Medicine, University of Leeds, Leeds, United Kingdom; ^3^ Core Research Laboratory, Department of Endocrinology, Department of Tumor and Immunology, Precision Medical Institute, Western China Science and Technology Innovation Port, The Second Affiliated Hospital, Health Science Center, Xi’an Jiaotong University, Xi’an, China; ^4^ NHS England & NHS Improvement (North East and Yorkshire), Leeds, United Kingdom

**Keywords:** ankle blood pressure, doppler, clinical blood pressure, glucose metabolism, OGTT (oral glucose tolerance test), pregnancy, race/ethnic differences

## Abstract

**Objective:**

This cross-sectional study aimed to determine the relationship between clinical blood pressures and blood pressures measured using Doppler with blood glucose in pregnancy by ethnicity.

**Methods:**

We recruited 179 (52% White European, 48% Asian) pregnant women at 24-28 weeks of gestation who underwent a glucose tolerance test in an antenatal clinic in Bradford Royal Infirmary, the UK, from 2012 to 2013. Systolic blood pressures in the arm (left and right brachial) and ankle [left and right posterior tibial (PT) and dorsalis pedalis (DP)] blood pressures were measured using a Doppler probe. The inter-arm (brachial) and inter-ankle (PT and DP) systolic blood pressure differences were obtained. A multivariate linear regression model adjusted for age, body mass index, and diabetes risk was used to assess the relationship between blood pressures and blood glucose.

**Results:**

Asian pregnant women had higher blood glucose but lower ankle blood pressures than White Europeans. In White Europeans, brachial blood pressures and clinical blood pressures were positively associated with fasting blood glucose (FBG), but brachial blood pressures did not perform better as an indicator of FBG than clinical blood pressures. In Asians, increased inter-ankle blood pressure difference was associated with increased FBG. For each 10 mmHg increase in the inter-ankle blood pressure difference, FBG increased by 0.12 mmol/L (Beta=0.12, 95%CI: 0.01-0.23).

**Conclusion:**

The relationship between blood pressures with blood glucose differed by ethnicity. In Asians, inter-ankle systolic blood pressure difference was positively associated with blood glucose. This is first ever report on ankle blood pressures with blood glucose in pregnancy which suggests future potential as a non-invasive gestational diabetes risk screening tool.

## Introduction

At the National Health Service Health Check in the UK, thresholds of body mass index (BMI) and blood pressures are used to screen for increased risk of cardio-metabolic diseases. However, blood pressure is not considered to calculate the risk of gestational diabetes mellitus (GDM). The current screening for the risk of GDM mainly depends on known risk factors such as age and BMI (1). Moreover, the current screening is applied to the general population and is not ethnicity-specific.

Asian pregnant women have a higher prevalence of GDM than White Europeans (2). They also have higher weight-related disease risks such as cardiovascular diseases at lower BMIs (3, 4). In this context, the method of screening high-risk populations for GDM based on known factors such as BMI may exclude Asian populations who have a lower BMI (5) and are not in the regular risk group. Therefore, it is crucial to develop an ethnicity-specific method to screen for GDM high-risk women.

We have previously demonstrated that the increased ankle blood pressures were associated with diabetes more than brachial blood pressures in non-pregnant primary care practice attendees (6). In addition, inter-ankle blood pressure difference has been demonstrated to better predict cardiovascular events in elderly patients compared with inter-arm blood pressure (7). So far, there is no study on the relationship between ankle blood pressure and blood glucose during pregnancy. Thus, this is the first study that aimed to determine the relationship between ankle blood pressure and its differences with blood glucose in pregnancy in Asians and White Europeans.

## Materials and Methods

### Study Population

In this cross-sectional study 184 pregnant women at 24-28 weeks of gestation were recruited at an antenatal clinic in Bradford Royal Infirmary, the UK, in 2012-2013. Consecutive pregnant women were recruited without selection bias. All pregnant women were screened for GDM with a 75 g oral glucose tolerance test (OGTT). GDM was diagnosed according to the recommendations of the National Institute for Health and Care Excellence criteria: fasting blood glucose (FBG) ≥ 5.6 mmol/L or 2-hour blood glucose (2h-BG) ≥ 7.8 mmol/L (8). Pregnant women older than 18 years who provided written informed consent were included. The exclusion criteria included: 1) pre-pregnancy type 1 or type 2 diabetes, 2) FBG≥7.0 mmol/L (n=1) (9), 3) refusal to consent, and 4) missing values for FBG and 2h-BG (n=4). Finally, 179 pregnant women were included. GDM diagnosis and testing criteria were the same during the study period. Pregnant women were approached only once, with glucose tolerance test and the measurements of blood pressure performed on the same day. This cross-sectional study followed the principles set by the Declaration of Helsinki, according to the Strengthening the Reporting of Observational Studies in Epidemiology (STROBE) guidelines and was approved by the National Research Ethics Committee (approval number: 10/H1302/28).

### Blood Pressure Measurement and Blood Pressure Differences

On the day of OGTT test, arm (left and right brachial) and ankle [left and right posterior tibial (PT) and dorsalis pedalis (DP)] systolic blood pressures were recorded using a Doppler probe (Huntleigh Super Dopplex II, Huntleigh Healthcare, Cardiff, UK) as we did previously (6, 10). The left ankle blood pressure was calculated as the average of left PT and DP, and the right ankle blood pressure was calculated in the same way.

Blood pressure and blood pressure differences were obtained Inter-arm (brachial), inter-PT, and inter-DP systolic blood pressure differences were calculated as the absolute value of the differences in brachial, PT and DP systolic blood pressure on the left and right sides, respectively. Inter-ankle systolic blood pressure difference was calculated as the absolute value of the differences in left and right ankle blood pressure.

### Data Collection

Patient clinical parameters collected from the medical records included age (years), ethnicity [White European, Asian (Indian, Pakistani, Bangladeshi, other Asian)], pregnancy week (weeks), BMI (kg/m^2^), diabetes family history, previous abnormal fasting glucose, abnormal glucose tolerance or GDM, clinical blood pressure (systolic and diastolic, mmHg). Pulse pressure (mmHg) was calculated as the difference between systolic and diastolic blood pressure. Hypertension was defined as systolic/diastolic BP≥140/90 mm Hg or treatment for hypertension.

### Statistical Analysis

Continuous variables were depicted as means and standard deviation and compared by *t-*test. Categorical variables were expressed as number (%) and compared by Chi-square test. Missing data were excluded from the analysis. We used linear regression to explore the relationship between blood pressure and blood glucose after adjusting for age, BMI and high diabetes risk (defined as family history of diabetes, previous abnormal fasting glucose, abnormal glucose tolerance or GDM). The statistical analyses were performed using the SPSS (IBM Corp. Released 2017. IBM SPSS Statistics for Windows, Version 25.0. Armonk, NY: IBM Corp.). A two-tailed *p*<0.05 was recognized statistically significant.

### Sensitivity Analysis

As GDM is more frequent in Asian population compared with White Europeans (11), it may influence the relationship of ankle and brachial blood pressure with blood glucose. Therefore, analyses were repeated following exclusion of GDM-positive women.

## Results

The participants included 179 pregnant women, 93 White Europeans and 86 Asians. The Asian group was comprised of 53 (61.6%) Pakistani, 24 (27.9%) Bangladeshi, 4 (4.6%) Indian and 5 (5.8%) women of other Asian origins. This group represents the Asian population in Bradford, the UK (12). Both groups had a similar gestational week, BMI, percentage of women with high diabetes risk (**Table 1**).

**Table 1:** Characteristics of study participants^a^.

	Entire population (n=179)			Population excluding GDM-positive women (n=163)		
	White Europeans (n=93)	Asians (n=86)	*P* ^b^	White Europeans (n=90)	Asians (n=73)	*P* ^b^
Age (years)	27.4 ± 5.0	28.9 ± 4.7	**0.045**	27.2 ± 4.8	28.2 ± 4.1	0.135
Gestational Weeks	26.4 ± 1.2	25.7 ± 2.9	0.171	26.3 ± 1.2	25.7 ± 2.4	0.120
Body mass index (kg/m^2^)	29.1 ± 5.5	28.5 ± 5.2	0.440	28.9 ± 5.3	28.3 ± 5.3	0.501
High Diabetes Risk ^c^	3 (3.2%)	4 (4.7%)	0.623	2 (2.2%)	2 (2.7%)	0.823
GDM cases	3 (3.2%)	13 (15.1%)	**0.005**	–	–	
***Blood glucose***						
Fasting blood glucose (mmol/L)	4.21 ± 0.45	4.46 ± 0.50	**0.001**	4.18 ± 0.38	4.37 ± 0.39	**0.002**
2-hour blood glucose (mmol/L)	5.26 ± 1.13	5.80 ± 1.65	**0.012**	5.17 ± 1.02	5.32 ± 1.03	0.379
***Systolic Blood pressure (Doppler)***						
***Arm***						
Left brachial (mmHg)	107.1 ± 12.0	102.6 ± 13.7	**0.024**	106.9 ± 12.1	102.0 ± 13.9	**0.020**
Right brachial (mmHg)	111.2 ± 12.7	106.6 ± 12.1	**0.014**	111.1 ± 12.8	106.7 ± 12.1	**0.032**
***Ankle***						
Left PT (mmHg)	128.1 ± 17.7	120.2 ± 19.0	**0.005**	127.7 ± 17.7	119.1 ± 19.8	**0.004**
Left DP (mmHg)	123.5 ± 22.7	114.7 ± 19.3	**0.007**	123.1 ± 22.9	113.3 ± 20.0	**0.005**
Left ankle (mmHg)	125.3 ± 18.7	117.3 ± 18.4	**0.005**	124.9 ± 18.8	116.0 ± 19.0	**0.004**
Right PT (mmHg)	127.7 ± 22.1	119.9 ± 19.4	**0.013**	127.5 ± 22.4	119.1 ± 19.9	**0.014**
Right DP (mmHg)	122.7 ± 23.0	116.7 ± 22.0	0.077	122.3 ± 23.2	115.5 ± 21.9	0.058
Right ankle (mmHg)	125.4 ± 20.5	118.4 ± 19.4	**0.022**	125.1 ± 20.7	117.4 ± 19.5	**0.019**
***Blood pressure difference***						
Inter-brachial difference (mmHg)	7.1 ± 7.4	8.1 ± 9.7	0.440	7.2 ± 7.5	8.2 ± 9.3	0.461
Inter-PT difference (mmHg)	9.8 ± 11.3	10.2 ± 10.4	0.804	9.9 ± 11.4	10.5 ± 11.0	0.721
Inter-DP difference (mmHg)	10.6 ± 13.4	11.5 ± 14.0	0.650	10.7 ± 13.6	11.4 ± 14.6	0.775
Inter-ankle difference (mmHg)	7.7 ± 7.8	10.0 ± 9.5	0.098	7.9 ± 7.9	10.2 ± 10.0	0.124
***Blood pressure (Clinics)***						
SBP (mmHg)	111.5 ± 13.8	109.1 ± 11.6	0.206	111.2 ± 13.9	108.9 ± 11.7	0.253
DBP (mmHg)	64.7 ± 11.3	62.3 ± 10.3	0.150	64.6 ± 11.4	62.7 ± 10.5	0.289
Pulse pressure (mmHg)	47.2 ± 13.3	46.8 ± 11.6	0.831	47.0 ± 13.4	46.2 ± 11.9	0.681

All (100%) of the records included information related to maternal age, ethnicity and high diabetes risk; 184 (99.5%) to body mass index, 183 (98.9%) to systolic and right dorsalis pedalis blood pressure; 182 (98.4%) to right brachial, left and right posterior tibial blood pressure; 180 (97.3%) to diastolic, left brachial, left dorsalis pedalis blood pressure, and 2-hour blood glucose; 174 (94.1%) to fasting blood glucose.

a Data are represented as means ± standard deviation;

b Student t-test was used to assess statistical significance;

c High diabetes risk was defined as a family history of diabetes or previous abnormal fasting glucose or abnormal glucose tolerance or GDM;

DBP, diastolic blood pressure; DP, dorsalis pedalis; GDM, gestational diabetes mellitus; n, number; PT, posterior tibial; n, number; SBP, systolic blood pressure. Significantly different p values are marked in bold.

### Ethnicity-Based Differences in Glycemic Control and Blood Pressures

There were significantly more GDM-positive cases among Asian pregnant women than White European pregnant women. Moreover, Asian participants were older, had higher FBG and 2h-BG. As a higher percentage of GDM-positive women among Asians may contribute to the observed differences in blood glucose concentrations, we excluded GDM patients and repeated the analysis. Asian pregnant women had a higher FBG, but not 2h-BG, than White European pregnant women (**Table 1**). These data suggest that White European and Asian pregnant women have different glucose metabolism as observed previously (13).

No difference was observed for routine clinically measured blood pressures. Two White European and one Asian women had hypertension. One Asian women was suffering from pre-eclampsia. Arm and ankle blood pressures (left PT, DP and ankle in general; right PT and ankle in general) measured using a Doppler probe were significantly higher in White European than Asian pregnant women (**Table 1**), similarly to what we observed previously in the non-pregnant populations (6, 10). These data suggest that Doppler examination of blood pressure in the ankle and arm arteries may be a more sensitive way to assess blood pressure status in pregnancy in different ethnicities.

As for the blood pressure difference, we observed that the lower the blood pressure measurement point in the body, the greater the difference in blood pressure between the left and right limbs. (Inter-DP > inter-PT > inter-brachial systolic blood pressure difference) in both groups. In terms of the comparison of the blood pressure difference between ethnic groups, Asians had higher inter-arm (brachial) and inter-ankle (PT, DP and overall) systolic blood pressure differences than White Europeans, but the differences did not reach significance.

### Relationship Between Blood Glucose and Blood Pressure in Pregnancy

We studied whether blood pressures might be related to blood glucose concentrations in pregnancy. Multiple linear regression analysis was conducted with blood pressure and blood glucose as continuous variables. Left brachial blood pressure (measured with Doppler) and systolic and diastolic blood pressure (routine clinical parameters) showed a positive association with FBG in White European pregnant women, also after excluding GDM patients. However, such a relationship was not observed in Asian pregnant women (**Table 2**).

**Table 2:** The association between blood pressures and fasting blood glucose by ethnicity.

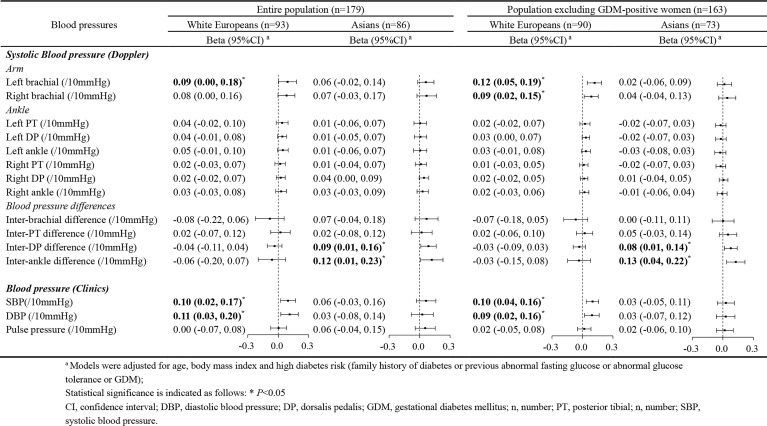

In Asians, fasting blood glucose was only related to the inter-ankle blood pressure difference, specifically inter-DP blood pressure difference. In general, each 10 mmHg rise in inter-ankle blood pressure difference, FBG increased by 0.12 mmol/L (95%CI: 0.01-0.23), and 0.13 mmol/L (95%CI: 0.04-0.22) after excluding GDM patients. Such a relationship was not found in White European study participants. These data indicate that inter-ankle systolic blood pressure difference can be developed as a surrogate for a non-invasive measure of glucose intolerance in Asian pregnant women.

No association between blood pressure and 2-h BG were found in Asians or White Europeans (**Supplementary Table**).

## Discussion

### Our Findings

We found that Asian pregnant women had higher blood glucose but lower ankle and brachial blood pressures (measured with Doppler) than White European pregnant women. In White European, left brachial and routine clinical blood pressure (SBP and DBP) were positively associated with FBG. While in Asian study participants, inter-ankle blood pressure difference was positively associated with FBG.

### Comparisons With Other Studies

The relationship between blood pressure/hypertension and GDM, which was characterized as increased blood glucose, has been reported in previous studies. Pregnancy with hypertension is a risk factor for GDM (14). Higher systolic and diastolic blood pressure (15) but similar mean arterial blood pressure (16) were reported in GDM-positive compared with GDM-negative women. Moreover, increased blood pressure in the first trimester increased the risk of GDM (17). Overall, we found that blood pressure was associated with FBG in Asians and White Europeans, but the association varied by ethnicity. In White Europeans, the association between brachial blood pressure and blood glucose was similar to that between clinical blood pressure and blood glucose, indicating that brachial blood pressure did not perform better as an indicator of blood glucose.

In Asians, the association between fasting blood pressure and blood glucose was only reflected in inter-ankle systolic blood pressure difference. Ankle blood pressure was reported to be a better discriminator than brachial blood pressure for diabetes, independent of age and sex (6). Furthermore, inter-ankle blood pressure difference was a more useful blood pressure parameter. Compared to inter-arm blood pressure difference, inter-ankle blood pressure difference could better predict cardiovascular events and all-cause mortality in elderly patients (7). Higher inter-ankle blood pressure difference was also crucial in predicting rapid renal progression and progression to renal endpoints in patients with chronic kidney disease (18). In this study, we found that the difference in blood pressure between DP was larger than the difference between the brachial and PT in all participants. Inter-ankle systolic blood pressure difference ≥ 10 mmHg was considered abnormal and not uncommon in Asians (19). We found that Asian pregnant women had higher inter-ankle systolic blood pressure difference than White Europeans; however, the differences did not reach significance due to the relatively small sample size. After adjusting for age, BMI and diabetes risk, we found that as the inter-ankle blood pressure difference (specifically DP difference) increased, the FBG concentrations increased in Asian pregnant women. Fasting blood glucose is the glucose concentration after an overnight fast that reveals mostly endogenous glucose production and indicates insulin resistance. Postprandial blood glucose represents a sharp increase in glucose in response to exogenous glucose, and 2-h BG glucose mainly indicates glucose clearance due to action of insulin (20). Therefore, ankle blood pressure could be indicative of pathological insulin resistance in pregnancy. The possible mechanism may be that a high inter-ankle blood pressure difference is a result of disproportional reduction in elasticity of arterioles between the right and the left lower limb due to insulin resistance (21), which precedes the increase in fasting blood glucose (22). When detected and treated early, these changes could be reversed with lifestyle modifications or insulin sensitizers. Moreover, blood glucose concentrations have been demonstrated as an independent positive factor for inter-ankle blood pressure differences (19).

The ethnic difference in the association between inter-ankle systolic blood pressure difference and blood glucose may be caused by genetic background. For example, our study found that Asian pregnant women had a higher blood glucose concentrations than White Europeans which is in concordance with reports of a higher prevalence of GDM (2). One possible reason is that Asians have higher visceral body fat than White Europeans of the same BMI (23, 24), which could result from a genetic predisposition for higher insulin resistance along with sedentary lifestyle. Insulin resistance causes changes to the structure and function of the arterioles and capillary systems, in the lower limbs (25–28) and hence, independent of systemic blood pressure. Although Asians have a higher risk of GDM, the current screening is done for the general population and is not ethnicity-specific. Our study provides evidence of the significance of ankle systolic blood pressure measurement as a surrogate sensitive marker of blood glucose in populations with a higher incidence of GDM and insulin resistance.

### Implications

Due to cost, OGTT is done only in high GDM risk populations in some regions (29). Current GDM risk screening is based on known risk factors such as age, BMI, diabetes family history but does not include blood pressure (1). Such selective GDM risk screening may exclude Asians, who have a lower BMI (5), so are not in the regular risk group. Therefore, it is crucial to develop an ethnicity-specific measures to screen for GDM high-risk pregnant women. Recently, abdominal visceral adipose tissue depth (VAD) measured by ultrasound in early pregnancy was proposed to predict GDM better than pre-pregnancy BMI and thus used in GDM screening (30). However, although VAD measurement is non-invasive, it is time-consuming and needs specialist training. On the contrary, ankle systolic blood pressure can be done easily by a community health care assistant, especially with automated machines. We propose that inter-ankle systolic blood pressure difference may serve as an early indicator of changes of increased insulin resistance and blood glucose in pregnancy and be developed as a sensitive, convenient and affordable method for GDM risk screening. Inter-ankle systolic blood pressure difference may also be developed to screen post-partum diabetes in GDM patients. Moreover, our findings can be further investigated in other ethnicities.

### Future Study

Brachial blood pressure changes dynamically during the various trimesters of pregnancy (31, 32). Whether similar changes can be observed in ankle blood pressure and what factors modulate these changes need elucidation. Thus, prospective studies starting in the first trimester with a larger group and different ethnicities need to be conducted to understand better the relationship between ankle blood pressure, its differences and blood glucose concentrations in pregnancy.

### Strengths and Limitations

pt?>To the best of our knowledge, we are the first to report that inter-ankle blood pressure difference is a marker of blood glucose in Asian pregnant women. Our study has several strengths. First, we included blood pressure measured by Doppler device and clinical blood pressure and obtained inter-arm and inter-ankle systolic blood pressure differences. The inclusion of multiple blood pressure indicators enables us to comprehensively explore the association between blood pressures and blood glucose during pregnancy. The second strength is the blind study design; experimentalists were unaware of the study subjects’ blood glucose concentrations. Third, GDM screening and testing criteria were the same during the study period; including measurements of blood pressure and blood glucose concentrations. Nevertheless, our study has some limitations. First, due to the cross-sectional study design, we cannot find a causal relationship between blood pressure and blood glucose. Second, the sample size is small, and analysis was done at a single center. Thus, we could not access the relationship between blood glucose and hypertension/preeclampsia. However, a significant association between inter-ankle blood pressure difference and blood glucose was found in this sample size, suggesting that our findings are sound.

## Conclusion

The relationship between blood pressure and blood glucose differed by ethnicity. Inter-ankle blood pressure difference was positively associated with fasting blood glucose concentrations independently of age, BMI and high diabetes risk in Asian pregnant women. Thus, inter-ankle systolic blood pressure difference may allow prediction or early detection of insulin-resistance-related changes in pregnancy, especially in non-White Europeans that are at an increased risk of developing GDM. Therefore, inter-ankle systolic blood pressure difference, together with known risk factors such as age and BMI, should be further developed as the early, convenient, non-expensive and non-invasive method to identify the high-risk of GDM for preventive interventions.

## Data Availability Statement

The datasets generated during and/or analysed during the current study are not publicly available since patient permission was not sought for the sharing of data, at the time of recruitment. Requests to access the datasets should be directed to k.kain@nhs.net. 

## Ethics Statement

The studies involving human participants were reviewed and approved by National Research Ethics Committee, the UK (REC reference number 10/H1302/28). The patients/participants provided their written informed consent to participate in this study.

## Author Contributions

The research conceptualization was by KK. KK designed research and supervised data acquisition; RZ, KK, and MG developed the analysis protocol and RZ analyzed the data; CC reviewed data analysis and discussed the project; MG, RZ and HV wrote the manuscript with the input from all the authors; all authors read and approved the final version of the manuscript.

## Funding

This research received no specific grant from any funding agency in public, commercial or not-for-profit sectors. Leeds Institute of Medical Education (LIME), University of Leeds UK sponsored the study and students who recruited the study subjects and collected the data. CC is a recipient of Mautner British Heart Foundation Career Development Fellowship. HV is sponsored by the British Heart Foundation Programme Grant. KK is a former Associate Professor at University of Leeds. MG is a recipient of the Start-up Budget from the Second Affiliated Hospital of Xi’an Jiaotong University (82668428). The Second Affiliated Hospital of Xi’an Jiaotong University supported RZ and the publication of the study.

## Conflict of Interest

The authors declare that the research was conducted in the absence of any commercial or financial relationships that could be construed as a potential conflict of interest.

## Publisher’s Note

All claims expressed in this article are solely those of the authors and do not necessarily represent those of their affiliated organizations, or those of the publisher, the editors and the reviewers. Any product that may be evaluated in this article, or claim that may be made by its manufacturer, is not guaranteed or endorsed by the publisher.
