# Supplementary material for: Inter-ankle Systolic Blood Pressure Difference Is a Marker of Increased Fasting Blood-Glucose in Asian Pregnant Women
Source: Front Endocrinol (Lausanne). 2022 May 31;13:842254. doi: 10.3389/fendo.2022.842254 (PMC9195077; doi:10.3389/fendo.2022.842254)
Supplement: Supplementary file 1 [file Table_1.docx]

**Supplementary Table. The association between blood pressures and 2-hour blood glucose by ethnicity**

| Blood pressures | Entire population (n=179) | |  | Population excluding GDM-positive women (n=163) | |
| --- | --- | --- | --- | --- | --- |
|  | White Europeans (n=93) | Asians (n=86) |  | White Europeans (n=90) | Asians (n=73) |
|  | Beta (95%CI) ^a^ | Beta (95%CI) ^a^ |  | Beta (95%CI) ^a^ | Beta (95%CI) ^a^ |
| ***Blood pressure (Doppler)*** |  |  |  |  |  |
| *Arm* |  |  |  |  |  |
| Left brachial (/10mmHg) | 0.18 (-0.06, 0.41) | 0.10 (-0.17, 0.36) |  | 0.15 (-0.08, 0.38) | 0.05 (-0.14, 0.23) |
| Right brachial (/10mmHg) | 0.08 (-0.14, 0.30) | 0.03 (-0.30, 0.35) |  | 0.07 (-0.14, 0.28) | 0.02 (-0.22, 0.25) |
| *Ankle* |  |  |  |  |  |
| Left PT (/10mmHg) | -0.01 (-0.16, 0.13) | -0.04 (-0.24, 0.17) |  | 0.00 (-0.14, 0.14) | -0.10 (-0.23, 0.04) |
| Left DP (/10mmHg) | 0.02 (-0.09, 0.13) | 0.08 (-0.12, 0.28) |  | 0.03 (-0.08, 0.13) | -0.04 (-0.17, 0.10) |
| Left ankle (/10mmHg) | 0.02 (-0.12, 0.15) | 0.02 (-0.20, 0.23) |  | 0.02 (-0.11, 0.15) | -0.08 (-0.23, 0.07) |
| Right PT (/10mmHg) | -0.05 (-0.17, 0.08) | -0.01 (-0.20, 0.17) |  | -0.04 (-0.16, 0.08) | -0.10 (-0.23, 0.03) |
| Right DP (/10mmHg) | -0.04 (-0.15, 0.07) | 0.04 (-0.12, 0.20) |  | -0.05 (-0.15, 0.05) | -0.04 (-0.16, 0.08) |
| Right ankle (/10mmHg) | -0.05 (-0.18, 0.08) | 0.00 (-0.19, 0.19) |  | -0.06 (-0.18, 0.07) | -0.09 (-0.22, 0.04) |
| *Blood pressure differences* |  |  |  |  |  |
| Inter-brachial difference (/10mmHg) | -0.21 (-0.57, 0.14) | -0.24 (-0.60, 0.13) |  | -0.16 (-0.51, 0.18) | 0.01 (-0.27, 0.30) |
| Inter-PT difference (/10mmHg) | -0.02 (-0.25, 0.20) | -0.20 (-0.54, 0.14) |  | 0.01 (-0.20, 0.23) | -0.07 (-0.30, 0.16) |
| Inter-DP difference (/10mmHg) | -0.11 (-0.29, 0.07) | 0.11 (-0.15, 0.36) |  | -0.08 (-0.25, 0.09) | 0.01 (-0.16, 0.18) |
| Inter-ankle difference (/10mmHg) | -0.29 (-0.60, 0.03) | 0.03 (-0.34, 0.41) |  | -0.21 (-0.51, 0.09) | -0.03 (-0.29, 0.22) |
|  |  |  |  |  |  |
| ***Blood pressure (Clinics)*** |  |  |  |  |  |
| SBP (/10mmHg) | 0.05 (-0.15, 0.25) | 0.04 (-0.28, 0.36) |  | 0.06 (-0.13, 0.25) | -0.04 (-0.27, 0.19) |
| DBP (/10mmHg) | 0.10 (-0.12, 0.33) | -0.04 (-0.40, 0.31) |  | 0.16 (-0.06, 0.37) | -0.07 (-0.32, 0.18) |
| Pulse pressure (/10mmHg) | -0.05 (-0.25, 0.15) | 0.07 (-0.25, 0.38) |  | -0.08 (-0.28, 0.11) | 0.01 (-0.21, 0.23) |
|  |  |  |  |  |  |

^a^ Models were adjusted for age, body mass index and high diabetes risk (family history of diabetes or previous abnormal fasting glucose or abnormal glucose tolerance or GDM);

CI, confidence interval; DBP, diastolic blood pressure; DP, dorsalis pedalis; GDM, gestational diabetes mellitus; n, number; PT, posterior tibial; n, number; SBP, systolic blood pressure.
